# Validation of the Brazilian Portuguese Version of the Western Ontario Shoulder Instability Index (WOSI) Questionnaire

**DOI:** 10.1055/s-0044-1790216

**Published:** 2024-12-07

**Authors:** Jose Carlos Souza Vilela, Tadeu Fonseca Barbosa, Daniel Oliveira Araujo, Yuri Vinicius Teles Gomes, Thalles Leandro Abreu Machado

**Affiliations:** 1 Cirurgia do Ombro e Cotovelo, Serviço de Ortopedia e Traumatologia, Hospital Unimed Belo Horizonte, Belo Horizonte, MG, Brasil; 2 Cirurgia do Ombro e Cotovelo, Hospital Madre Teresa, Belo Horizonte, MG, Brasil; 3 Fundação Hospitalar do Estado de Minas Gerais (FHEMIG), Belo Horizonte, MG, Brasil; 4 Cirurgia de Ombro e Cotovelo, Hospital Governador Israel Pinheiro (HGIP), Instituto de Previdência dos Servidores do Estado de Minas Gerais (Ipsemg), Belo Horizonte, MG, Brasil

**Keywords:** arthroscopy, joint instability, shoulder joint, sports medicine, surveys and questionnaires, translations

## Abstract

**Objective** To evaluate the validity of the Brazilian Portuguese version of the Western Ontario Shoulder Instability Index (WOSI).

**Methods** We assessed 51 patients aged 18 to 40 years who were divided into 3 groups: 17 patients with shoulder instability undergoing conservative treatment, 17 with shoulder instability treated surgically, and 17 without shoulder instability. The patients underwent functional and health assessments using the following scores: WOSI, Rowe, Visual Analog Scale (VAS), Subjective Shoulder Value (SSV), Disabilities of the Arm, Shoulder and Hand (DASH), and University of California-Los Angeles (UCLA) Shoulder Scale.

**Results** The variables sex and age were homogeneous among the groups ( *p* > 0.05). A comparative analysis of the scores revealed that patients undergoing treatment (either surgical or conservative) showed significant differences compared with the control group ( *p* < 0.05). The determination of score correlation was made using the Spearman correlation coefficient. All instruments analyzed showed a significant relationship among themselves but at different levels: the correlation between the WOSI and DASH instruments was perfectly positive ( *r* = 0.96); the comparison of the WOSI and UCLA ( *r* = 0.87), DASH and UCLA ( *r* = 0.86), SSV and Rowe ( *r* = 0.80), VAS and DASH (r = 0.75), VAS and UCLA ( *r* = 0.74), and WOSI and VAS ( *r* = 0.72) also showed a trend towards positive linearity among measurements; and the comparison of the instruments WOSI and SSV, WOSI and Rowe, DASH and Rowe, SSV and UCLA ( *r* = -0.83), SSV and DASH ( *r* = -0.79), Rowe and UCLA ( *r* = -0.78), VAS and SSV ( *r* = -0.68), and VAS and Rowe ( *r* = -0.60) revealed a negative correlation.

**Conclusion** The Brazilian Portuguese version of the WOSI presents good validity.

## Introduction

Complaints of shoulder abnormalities are frequent in the orthopedic practice. The shoulder can present clinical manifestations with painful complaints of varying degrees and episodes of instability. 1 The particular biomechanics of the shoulder partly explain these abnormalities, since daily and sporting activities require a high shoulder joint range of motion and mechanical demands. Shoulder instability is a common issue that mainly affects patients in the second and third decades of life. 2 The incidence of traumatic dislocation is of approximately 1.7% in the general population. 3 After the first episode, symptoms of chronic shoulder instability may appear with variable intensity and frequency. Although pain may be present, it is not the main symptom. The predominant complaints are apprehension and loss of confidence, resulting in reduced participation in sporting activities and a general decrease in quality of life. 4 5

Many treatments have been proposed for different types of instability; however, few assessment instruments prove the effectiveness of these treatments. 2 The clinical examination alone does not adequately reflect the functional impairments experienced by patients. Thus, a subjective analysis of the patient1s quality of life becomes a significant criterion. 4

Initially, the best method to evaluate posttreatment outcomes was observation. However, observation ignores the patient's perception of their health condition. Several studies 6 have demonstrated that objective functional assessments of the shoulder can be inconsistent and susceptible to errors.

Thus, self-administered questionnaires have been recommended as an effective method to assess the functional status and perception of symptoms by the patient, valuing their opinion about their health condition. 7

Different scores, indices, and scales evaluate the functional impact of shoulder conditions and health in general. These tools comprise generic assessment (such as the 36-Item Short Form Health Survey, SF-36), 8 limb-specific (such as the Disabilities of the Arm, Shoulder, and Hand [DASH] questionnaire and its shortened version, the QuickDASH), 9 shoulder-specific (such as the American Shoulder and Elbow Surgeons Score [ASES], the Constant-Murley score, the University of California-Los Angeles [UCLA] Shoulder Scale, the Subjective Shoulder Value [SSV]), 10 and condition-specific instruments (such as the Rowe score, the Western Ontario Shoulder Instability Index [WOSI], the Western Ontario Rotator Cuff [WORC], the Melbourne Instability Shoulder Scale [MISS], and the Oxford Shoulder Instability Questionnaire [OSIQ]). 6 11 12 13

Specific self-administered questionnaires for shoulder instability have been developed recently, such as the WOSI, OSIQ, and MISS. However, the MISS has shown less satisfactory psychometric properties than the WOSI. 4

The WOSI is simpler, more effective, and more reproducible compared with other instruments. Due to these characteristics, many consider it the best assessment method for patients with shoulder instability. 7 14 Furthermore, it is easy to use (with an estimated completion time of 3 minutes), reliable, reproducible, sensitive to changes, and it has undergone validation in multiple languages. 2 4 5 7 15 16 17 18 19 20 21 22

The WOSI is a quality-of-life questionnaire developed for use in English. The study of its psychometric properties showed a strong correlation with the DASH and the UCLA instruments. In the assessment of reproducibility, the intraclass correlation coefficient was excellent. It was developed and validated to be applied to patients with shoulder instability. As a specific instrument, it encompasses aspects of quality of life relevant to this disease. It contains 21 questions covering 4 domains: 1) physical symptoms; 2) sports, recreation, and work; 3) lifestyle; and 4) emotional status. 2 5

The answers to the WOSI questions are provided through the Visual Analog Scale (VAS). All questions have the same weight value. Therefore, each item may receive a score from 0 to 100 on the VAS, and the final result ranges from 0 to 2,100. The closer the final result is to the lower limit, the lower the disease impact and the less significant the change in quality of life. 5

The development of translation and cultural adaptation methods have enabled the use of an instrument developed in a given language and culture in another language and another cultural context after translation and adaptation. 23 24 Barbosa et al. 5 performed this process in 2012, obtaining the Brazilian Portuguese version of the questionnaire. 5 13

The validation stage consists of checking whether the new instrument retained the characteristics of the original version. This entire process is relevant so that the new tool is equivalent to the original version and culturally accepted in the country in question. 5

Therefore, although the original WOSI already had its psychometric properties studied, the objective of the present study is to evaluate its validity in the Brazilian Portuguese version.

## Materials and Methods

### Patient Selection

The inclusion criteria were the following: Patients who attended the shoulder and elbow outpatient clinic at Hospital Unimed Belo Horizonte, in the state of Minas Gerais, Brazil, and underwent an assessment for regular follow-up after treatment for shoulder instability. We divided these subjects into those receiving surgical and conservative treatments. The control group consisted of patients with other shoulder conditions but no instability and volunteers without orthopedic shoulder complaints. We excluded patients who chose not to sign the informed consent form (ICF), and those unable to fill out the forms due to psychiatric or psychological issues, neurological conditions, systemic inflammatory diseases, neoplasms, or cervical radiculopathy. All patients participating in the study signed the ICF.

Sample calculation followed the principles set out by Cochran 25 and Pereira. 26 The Cochran 25 formula calculates an ideal sample with predetermined precision levels (margin of error) and confidence intervals. For our population, we chose a 95% confidence interval (95%CI) and a 5% margin of error. Pereira's 26 formula assesses the sample size in linear correlation studies, as in the current study. Per this author, 26 the sample calculation employs the Spearman correlation coefficient. For the present study, we used the coefficient reported by Perrin et al., 4 who validated the WOSI for French using scores similar to those analyzed by our group. We found a maximum variation of three patients for the sample calculation employing the different variables under analysis (scores). Therefore, as there is more than one variable in the current study, we used the highest value derived from the sample calculation, finding a minimum number of 17 patients per group and totaling 51 subjects. We separated the patients into three groups:

- Conservative group, consisting of 17 patients with shoulder instability who did not undergo surgical treatment;
- Postoperative group, consisting of 17 patients with shoulder instability who underwent surgical treatment with a minimum follow-up of 12 months; and
- Control group, with 17 patients without shoulder instability.

### Data Collection

Two orthopedists specializing in shoulder and elbow surgery assessed all the patients and applied the following functional and health assessment scores: WOSI, Rowe, VAS, SSV, DASH, and UCLA ( Appendix 1 ).

## Results

We evaluated 51 patients, including 34 with anterior shoulder instability, 17 undergoing surgical treatment, and 17 undergoing conservative treatment. The other 17 subjects from the control group did not have shoulder instability.

Table 1 presents the demographic variables sex and age. The groups were homogeneous, with no significant difference among them ( *p* > 0.05).

**Table 1:** Characterization of the study sample per sex and age

	Sex: n (%)		*p* -value
	Male	Female	
**Postoperative group (n = 17)**	15 (88.24%)	2 (11.76%)	0.8553 ^T^
**Conservative group (n = 17)**	15 (88.24%)	2 (11.76%)	
**Control group (n = 17)**	14 (86.28%)	3 (13.72%)	
**Total (n = 51)**	44 (82.36%)	7 (17.64%)	
	**Age (years): mean(± standard deviation)**		***p*** **-value**
**Postoperative group (n = 17)**	26.88(± 6.33)		0.2391 ^T^
**Conservative group (n = 17)**	29.12(± 5.20)		
**Control group (n = 17)**	26.0 (± 4.42)		
**Total (n = 51)**	27.35(± 5.43)		

**Note:**^T^ Tukey test, suitable for data with parametric distribution

The comparative score analysis occurred as follows: normality test (Shapiro-Wilk and Kolmogorov-Smirnov), median and percentile measurements (25th and 75th percentiles – P25 and P75 respectively), and statistical data analysis (Kruskal-Wallis test followed by the Dunn test).

Regardless of the score, the postoperative and conservative groups showed significant differences compared with the control group ( *p* < 0.05), except for the postoperative group in the Rowe score.

Only the VAS score was unable to demonstrate a statistical difference when comparing the postoperative and conservative groups ( *p* > 0.05) ( Table 2 ).

**Table 2:** Comparison of the WOSI, VAS, SSV, Rowe, DASH, and UCLA scores of the sample on the instruments used

	Posperative (n = 17)	Conservative (n = 17)	Control (n = 17)	Total (n = 51)	
	Median (25P–75P)	Median (25P–75P)	Median (25P–75P)	Median (25P–75P)	*p* -value
**WOSI**	425.0* ^$^ (112.5–97.5)	1000.0* (870.0–348.0)	0.0 (0.0–122.5)	360.0 (10.0–340.0)	< 0.0001 ^KW^
**VAS**	2.0* (0.0–4.0)	4.0* (1.5–6.0)	0.0 (0.0–1.0)	1.0 (0.0–4.0)	0.0004 ^KW^
**SSV**	80.0* ^$^ (55.0–97.5)	50.0* (45.0–65.0)	100.0 (99.0–100.0)	80 (50.0–100.0)	< 0.0001 ^KW^
**Rowe**	100.0 ^$^ (85.0–100.0)	50.0* (40.0–75.0)	100.0 (99.0–100.0)	95.0 (55.0–100.0)	< 0.0001 ^KW^
**DASH**	7.5* ^$^ (1.3–13.8)	25.8* (17.5–38.8)	0.0 (0.0–2.1)	7.5 (0.0–19.2)	< 0.0001 ^KW^
**UCLA**	33.0* ^$^ (29.0–35.0)	29.0* (22. –29.0)	35.0 (35.0–35.0)	33.0 (29.0–35.0)	< 0.0001 ^KW^

**Abbreviations:** 25P, 25th percentile; 75P, 75th percentile; DASH, Disabilities of the Arm, Shoulder and Hand; SSV, Subjective Shoulder Value; VAS, Visual Analog Scale; UCLA, University of California-Los Angeles; WOSI, Western Ontario Shoulder Instability Index.

**Notes:** *Significantly different than the control group. ^$^ Significantly different than the conservative group. ^KW^ Kruskal-Wallis test (suitable for non-parametric data comparison).

The correlation among scores was assessed through the Spearman correlation coefficient, which evaluates the intensity degree in the relationship between two non-parametric variables. This coefficient correlation ranges from -1 and +1, and the higher the absolute value, the stronger the relationship among scores.

All instruments presented a significant relationship among themselves according to Spearman coefficient ( *p* < 0.05). However, the correlation level was different, as follows:

- The correlation was perfectly positive (with an *r* -value close to 1) for the WOSI and the DASH instruments ( *r* = 0.96). This result indicates that the measurements from both indicators increase significantly at the same intensity.
- The comparison of the WOSI and UCLA ( *r* = 0.87), DASH and UCLA ( *r* = 0.86), SSV and Rowe ( *r* = 0.80), VAS and DASH ( *r* = 0.75), VAS and UCLA ( *r* = 0.74), and WOSI and VAS ( *r* = 0.72) also showed a trend towards positive linearity between measurements.
- The correlation of the WOSI and SSV, WOSI and Rowe, DASH and Rowe, SSV and UCLA ( *r* = -0.83), SSV and DASH ( *r* = -0.79), Rowe and UCLA ( *r* = -0.78), VAS and SSV ( *r* = -0.68), and VAS and Rowe ( *r* = -0.60) was negative .

## Discussion

The assessment and analysis of the outcomes of certain medical treatments have evolved in recent times. The patient's perception of their problem has become more valued in the current context. Thus, scores relying on a more subjective analysis have become the most recommended methods.

Although there are other assessment methods for shoulder instability, the WOSI has been considered the best one, 7 14 and it has already been validated in several languages. 2 4 5 7 15 16 17 18 19 20 21 22 The choice of the analysis method for the current study relied on previously published articles validating this score.

The selected scores considered the potential comparison with other WOSI validation studies and encompass different assessment instruments, whether generic (VAS), limb-specific (DASH), shoulder-specific (UCLA, SSV), or condition-specific (Rowe). The DASH has been validated in Brazilian Portuguese, and the UCLA 10 and Rowe 11 scores have undergone translation and cultural adaptation to Brazilian Portuguese. The use of the VAS use in its Brazilian Portuguese version is widespread despite the lack of a published specific validation. We attribute this to the fact that it is easily understandable, since it is a visual scale, with no complex questions that would depend on cultural adaptation. Likewise, the SSV is a subjective assessment performed by the patient and expressed as a percentage, using 100% as a reference value for a completely normal shoulder.

All patients in the postoperative and conservative groups presented anterior instability (to reduce bias in result interpretation). The average age of the study patients (of 27.35 ± 5.43 years) and the sex distribution (44 men [82.36%] and 7 women [17.64%]) were consistent with that of previous studies 4 7 18 and homogeneous at group comparison ( Table 1 ).

The analysis of the validity of the WOSI in Brazilian Portuguese, according to Spearman coefficient, revealed a significant good or excellent correlation among all scores (presenting values close to -1 or 1). This coefficient is classified as excellent (> 0.91), good (0.90–0.71), fair (0.70–0.51), poor (0.50–0.31), and null (<c0.31). 4

Individually, the WOSI presents an almost a perfect correlation with the DASH score (0.96) and a high correlation with the UCLA (0.87) and VAS (0.72) scores ( Table 3 ). These results are consistent with those of the original article (DASH = 0.77; UCLA = 0.65) 2 regarding the French (QuickDASH = 0.78; VAS = 0.83), 4 Turkish (DASH = 0.67), 7 German (UCLA = 0.61), 21 Dutch (DASH = 0.81), 20 Swedish (VAS = 0.80), 15 Italian (DASH = 0.79), 18 and Japanese (QuickDASH = 0. 63) validations. 17 All showed a positive linearity for such scores, especially the DASH and UCLA.

**Table 3:** Correlation analysis (Spearman coefficient) of the scores on the WOSI, VAS, SSV, Rowe, DASH, and UCLA instruments

		WOSI	VAS	SSV	Rowe	DASH	UCLA
**WOSI**	*r* -value		0.72	-0.83	-0.83	0.96	0.87
	*p* -value		< 0.0001	< 0.0001	< 0.0001	< 0.0001	< 0.0001
**VAS**	*r* -value	0.72		-0.68	-0.60	0.75	0.74
	*p* -value	< 0.0001		< 0.0001	< 0.0001	< 0.0001	< 0.0001
**SSV**	*r* -value	-0.83	-0.68		0.80	-0.79	-0.83
	*p* -value	< 0.0001	< 0.0001		< 0.0001	< 0.0001	< 0.0001
**Rowe**	*r* -value	-0.83	-0.60	0.80		-0.83	-0.78
	*p* -value	< 0.0001	< 0.0001	< 0.0001		< 0.0001	< 0.0001
**DASH**	*r* -value	0.96	0.75	-0.79	-0.83		0.86
	*p* -value	< 0.0001	< 0.0001	< 0.0001	< 0.0001		< 0.0001
**UCLA**	*r* -value	0.87	0.74	-0.83	-0.78	0.86	
	*p* -value	< 0.0001	< 0.0001	< 0.0001	< 0.0001	< 0.0001	

**Abbreviations:** DASH, Disabilities of the Arm, Shoulder and Hand; SSV, Subjective Shoulder Value; VAS, Visual Analog Scale; UCLA, University of California-Los Angeles; WOSI, Western Ontario Shoulder Instability Index.

The Rowe score correlation (-0.83) presented good negative linearity, similar to the French (-0.54) 4 and Turkish (-0.57) 7 analysis, and the original (0. 61), 2 German (0.62), 21 and Swedish papers (0.59), 15 but with positive linearity. The SSV presented good negative linearity (-0.83), but no comparison with other studies.

The positive points of the present work include the number of analyzed scores (five, higher than other validation studies using three or four scores), group homogeneity, the representation of shoulder instability epidemiology, and the high significant correlation among the scores. The negative points include the small but significant sample size and the failure to perform test-retest and interobserver analysis.

## Conclusion

We concluded that the Brazilian Portuguese version of the WOSI presents good validity.
