# Supplementary material for: Validation of the Brazilian Portuguese Version of the Western Ontario Shoulder Instability Index (WOSI) Questionnaire
Source: Rev Bras Ortop (Sao Paulo). 2024 Dec 7;59(5):e765–70. doi: 10.1055/s-0044-1790216 (PMC11624921; doi:10.1055/s-0044-1790216)
Supplement: Related texts

# Research article: Validação da versão em português brasileiro do questionário Western Ontario Shoulder Instability Index (WOSI)

**Keywords:** articulação do ombro, artroscopia, inquéritos e questionários, instabilidade articular, medicina esportiva, traduções

## Abstract

**Objetivo** Avaliar a validade da versão em português do brasil do Western Ontario Shoulder Instability Index (WOSI).

**Métodos** Foram avaliados 51 pacientes, com idades entre 18 e 40 anos, que foram separados em 3 grupos: 17 pacientes com instabilidade do ombro em tratamento conservador, 17 com instabilidade do ombro tratados cirurgicamente e 17 sem instabilidade do ombro. Os pacientes foram submetidos a avaliações funcional e de saúde pelos seguintes escores: WOSI, Rowe, Escala Visual Analógica (EVA), Subjective Shoulder Value (SSV), Disabilities of the Arm, Shoulder and Hand (DASH), e University of California-Los Angeles (UCLA) Shoulder Scale.

**Resultados** As variáveis sexo e idade se mostraram homogêneas entre os grupos ( *p* > 0,05). Após análise comparativa dos escores, os pacientes submetidos a tratamento (cirúrgico ou conservador) apresentaram diferenças significativas na comparação com o grupo de controle ( *p* < 0,05). A correlação entre os escores foi avaliada submetendo as medidas ao coeficiente de correlação de Spearman. Todos os instrumentos analisados apresentaram relação significativa entre si. Entretanto, o nível de correlação foi distinto: foi observada correlação positiva perfeita entre os instrumentos WOSI e DASH ( *r* = 0,96); os instrumentos WOSI e UCLA ( *r* = 0,87), DASH e UCLA ( *r* = 0,86), SSV e Rowe ( *r* = 0,80), EVA e DASH ( *r* = 0,75), EVA e UCLA ( *r* = 0,74) e WOSI e EVA ( *r* = 0,72) também apresentaram tendência de linearidade positiva entre as medidas; já os instrumentos WOSI e SSV, WOSI e Rowe, DASH e Rowe, SSV e UCLA ( *r* = -0,83), SSV e DASH ( *r* = -0,79), Rowe e UCLA ( *r* = -0,78), EVA e SSV ( *r* = -0,68) e EVA e Rowe ( *r* = -0,60) apresentaram correlação negativa.

**Conclusão** A versão do WOSI em português do Brasil apresenta boa validade.

## Introdução

As queixas de alterações no ombro estão entre as mais frequentes na prática ortopédica. O ombro pode apresentar manifestações clínicas com queixas dolorosas de graus variados e episódios de instabilidade, 1 em parte explicados pela sua biomecânica particular, com grande amplitude articular e solicitações mecânicas exigidas nas atividades cotidianas e esportivas. A instabilidade do ombro é um problema comum, e afeta principalmente pacientes na segunda e terceira décadas de vida. 2 Na população geral, a luxação traumática tem incidência aproximada de 1,7%. 3 Após o primeiro episódio, podem surgir sintomas de instabilidade crônica de intensidade e frequência variáveis. A dor pode estar presente, mas não é o principal sintoma. A queixa predominante é apreensão e perda de confiança, que implicam redução na participação de atividades esportivas e decréscimo geral na qualidade de vida. 4 5

Muitos tratamentos foram propostos para os diversos tipos de instabilidade; entretanto, existem poucos instrumentos de avaliação que comprovem a eficácia dos tratamentos realizados. 2 Isoladamente, o exame clínico não reflete adequadamente os impedimentos funcionais vivenciados pelos pacientes. Assim, uma análise subjetiva do próprio paciente sobre sua qualidade de vida se torna um critério importante. 4

A princípio, o método de avaliação baseado na análise do observador era considerado a melhor opção para a mensuração dos resultados após o tratamento. Esta prática, porém, ignora a percepção do próprio paciente sobre sua condição de saúde. Vários estudos 6 têm demonstrado que a avaliação funcional objetiva do ombro pode ser inconsistente, e mais susceptível a erros.

Dessa forma, tem se recomendado o uso de questionários autoadministrados como método eficaz de mensuração que permite a avaliação do *status* funcional e da percepção dos sintomas pelo paciente, e valoriza sua opinião sobre sua condição de saúde. 7

Diferentes escores, índices e escalas foram projetados para avaliar a repercussão funcional das patologias do ombro e da saúde em geral; eles se dividem em instrumentos de avaliação genéricos (como o 36-Item Short Form Health Survey, SF-36), 8 específicos do membro (como o Disabilities of the Arm, Shoulder and Hand [DASH] e sua versão curta, QuickDASH), 9 específicos do ombro (como o American Shoulder and Elbow Surgeons Score [ASES], o de Constant-Murley, a University of California-Los Angeles [UCLA] Shoulder Scale, e o Subjective Shoulder Value [SSV]) 10 e específicos para determinada patologia (como o de Rowe, o Western Ontario Shoulder Instability Index [WOSI], o Western Ontario Rotator Cuff Index [WORC], a Melbourne Instability Shoulder Scale [MISS], e o Oxford Shoulder Instability Questionare [OSIQ]). 6 11 12 13

Recentemente, questionários autoadministrados específicos para instabilidade do ombro tem sido desenvolvidos, como o WOSI, o OSIQ e a MISS, mas estes últimos mostraram propriedades psicométricas menos satisfatórias do que o WOSI. 4

O WOSI é mais simples, mais eficaz e mais reprodutível quando comparado a outros instrumentos. Por essas características, ele é considerado o melhor método de avaliação de pacientes com instabilidade do ombro. 7 14 Além disso, é fácil de ser usado (tem preenchimento estimado em 3 minutos), confiável, reprodutível, sensível a mudanças, e tem sido validado em vários idiomas. 2 4 5 7 15 16 17 18 19 20 21 22

O WOSI é um questionário de qualidade de vida desenvolvido em língua inglesa. O estudo de suas propriedades psicométricas mostrou forte correlação com os instrumentos DASH e UCLA, e, na avaliação da reprodutibilidade, o coeficiente de correlação intraclasse foi considerado excelente. Foi criado e validado para ser aplicado em pacientes com instabilidade do ombro. Sendo um instrumento específico, engloba aspectos de qualidade de vida relevantes para essa doença. Contém 21 questões, que abrangem 4 domínios: 1) sintomas físicos; 2) esportes, recreação e trabalho; 3) estilo de vida; e 4) estado emocional. 2 5

As respostas às questões do WOSI são dadas por meio da Escala Visual Analógica (EVA). Todas as questões têm o mesmo valor ponderal. Portanto, cada item pode ser pontuado de 0 a 100 na EVA, e o resultado final pode variar de 0 a 2.100. Quanto mais o resultado final se aproxima do limite inferior, menor é o impacto da doença e menos significante é a alteração na qualidade de vida. 5

O desenvolvimento de métodos de tradução e adaptação cultural possibilitou que um instrumento desenvolvido para ser usado em determinada língua e cultura possa também ser usado, após tradução e adaptação, em outra língua e em outro contexto cultural. 23 24 Esse processo foi realizado por Barbosa et al. 5 em 2012, que desenvolveram a versão brasileira do questionário. 5 13

A fase de validação consiste em verificar se o novo instrumento manteve as características da versão original. Todo esse processo é relevante para que o instrumento seja equivalente à versão original e culturalmente aceito no país em questão. 5

Sendo assim, apesar do WOSI original já ter suas propriedades psicométricas estudadas, o objetivo deste estudo é avaliar sua validade na versão em português do Brasil.

## Materiais e Métodos

## Seleção dos Pacientes

Critérios de inclusão: foram avaliados pacientes que compareceram ao ambulatório de ombro e cotovelo do Hospital Unimed Belo Horizonte, para seguimento habitual após tratamento de instabilidade do ombro; eles foram divididos em pacientes submetidos a tratamento cirúrgico e pacientes submetidos a tratamento conservador. Para a formação do grupo controle, foram avaliados tanto pacientes com outras patologias do ombro, sem instabilidade, quanto voluntários sem queixas ortopédicas nos ombros. Foram excluídos os pacientes que optaram por não assinar o termo de consentimento livre e esclarecido (TCLE), bem como os pacientes com incapacidade de preencher os formulários por questões psiquiátricas ou psicológicas, problemas neurológicos, doenças inflamatórias sistêmicas, neoplasias ou radiculopatia cervical. Todos os pacientes que participaram do estudo assinaram o TCLE.

O cálculo da amostra foi obtido com o auxílio dos cálculos disponibilizados por Cochran 25 (1967) e Pereira 26 (2005),. A fórmula de Cochran 25 permite o cálculo de amostra ideal com níveis predeterminados de precisão (margem de erro) e intervalo de confiança. Para a nossa população, utilizou-se intervalo de confiança de 95% (IC95%) e margem de erro de 5%. A fórmula de Pereira 26 é utilizada na avaliação de tamanho de amostras de estudos de correlação linear, como é o caso deste estudo. O cálculo amostral segundo Pereira 26 utiliza o coeficiente de correlação de Spearman, e, para este estudo, utilizamos o coeficiente encontrado no estudo de Perrin et al., 4 que realizaram a validação do WOSI para o francês, e utilizaram escores semelhantes aos analisados por nosso grupo. Encontramos variação máxima de 3 pacientes para o cálculo amostral utilizando as diferentes variáveis a serem analisadas (os escores). Assim sendo, como havia mais de uma variável a ser estudada, utilizamos como cálculo amostral o maior valor encontrado nesses cálculos: um número mínimo de 17 pacientes por grupo, num total 51 pacientes avaliados. Os pacientes foram separados em três grupos:

- Grupo conservador, constituído de 17 pacientes com instabilidade do ombro não submetidos a tratamento cirúrgico;
- Grupo pós-operatório, constituído de 17 pacientes com instabilidade do ombro tratados cirurgicamente e com seguimento mínimo de 12 meses; e
- Grupo de controle, com 17 pacientes sem instabilidade do ombro.

## Coleta de Dados

Todos os pacientes foram avaliados por dois ortopedistas, especializandos em cirurgia do ombro e cotovelo, e submetidos a avaliações funcional e de saúde pelos seguintes escores: WOSI, ROWE, EVA, SSV, DASH e UCLA ( Apêndice 1 ).

## Resultados

Foram avaliados 51 pacientes, 34 com instabilidade anterior do ombro, sendo 17 submetidos a tratamento cirúrgico e 17, a tratamento conservador. Os outros 17 do grupo de controle não tinham instabilidade no ombro.

As variáveis demográficas sexo e idade foram analisadas e apresentadas na Tabela 1 . Os grupos se mostraram homogêneos, sem diferença significativa entre si ( *p* > 0,05).

**Tabela 1:** Caracterização da amostra do estudo segundo sexo e idade

	Sexo: n (%)		Valor de *p*
	Masculino	Feminino	
**Grupo pós-operatório (n = 17)**	15 (88,24%)	2 (11,76%)	0,8553 ^T^
**Grupo conservador (n = 17)**	15 (88,24%)	2 (11,76%)	
**Grupo de controle (n = 17)**	14 (86,28%)	3 (13,72%)	
**Total (n = 51)**	44 (82,36%)	7 (17,64%)	
	**Idade (anos): média(± deesvio padrão)**		**Valor de** ***p***
**Grupo pós-operatório (n = 17)**	26,88(± 6,33)		0,2391 ^T^
**Grupo conservador (n = 17)**	29,12(± 5,20)		
**GRUPO de controle (n = 17)**	26,06(± 4,42)		
**Total (n = 51)**	27,35(± 5,43)		

**Nota:**^T^ Teste de Tukey, adequado para dados com distribuição paramétrica.

A análise comparativa entre os escores foi realizada por meio de testes de normalidade (Shapiro-Wilk e Kolmogorov-Smirnov), medidas de mediana e percentil (percentis 25 e 75, P25 e P75, respectivamente) e análise estatística dos dados obtidos (teste de Kruskal-Wallis, seguido do teste de Dunn).

Independente do escore utilizado, os grupos pós-operatório e conservador apresentaram diferenças significativas na comparação com o grupo de controle ( *p* < 0,05), com exceção do grupo pós-operatório no escore de Rowe.

Somente a pontuação na EVA não foi capaz de demonstrar diferença estatística na comparação entre os grupos pós-operatório e conservador ( *p* > 0,05) ( Tabela 2 ).

**Tabela 2:** Comparação das das pontuações da amostra nos instrumentos utilizados

	Pós-operatório (n = 17)	ConservadorOR (n = 17)	Controle (n = 17)	Total (n = 51)	
	Mediana (25P–75P)	Mediana (25P–75P)	Mediana (25P–75P)	Mediana (25P–75P)	Valor de *p*
**WOSI**	425,0* ^$^ (112,5–97,5)	1000,0* (870,0–348,0)	0,0 (0,0–122,5)	360,0 (10,0–340,0)	< 0.0001 ^KW^
**EVA**	2,0* (0,0–4,0)	4,0* (1,5–6,0)	0,0 (0,0–1,0)	1,0 (0,0–4,0)	0,0004 ^KW^
**SSV**	80,0* ^$^ (55,0–97,5)	50,0* (45,0–65,0)	100,0 (99,0–100,0)	80 (50,0–100,0)	< 0.0001 ^KW^
**Rowe**	100,0 ^$^ (85,0–100,0)	50,0* (40,0–75,0)	100,0 (99,0–100,0)	95,0 (55,0–100,0)	< 0.0001 ^KW^
**DASH**	7,5* ^$^ (1,3–13,8)	25,8* (17,5–38,8)	0,0 (0,0–2,1)	7,5 (0,0–19,2)	< 0.0001 ^KW^
**UCLA**	33,0* ^$^ (29,0–35,0)	29,0* (22,0–29,0)	35,0 (35,0–35,0)	33,0 (29,0–35,0)	< 0.0001 ^KW^

**Abreviaturas:** 25P, percentil 25; 75P, percentil 75; DASH, Disabilities of the Arm, Shoulder and Hand; EVA, Escala Visual Analógica; SSV, Subjective Shoulder Value; UCLA, University of California-Los Angeles Shoulder Scale; WOSI, Western Ontario Shoulder Instability Index.

**Notas:** *Diferença significativa em relação ao grupo de controle. ^$^ Diferença significativa em relação ao grupo conservador. ^**KW**^ Teste Kruskal-Wallis, adequado para comparação de dados não paramétricos.

A correlação entre os escores foi avaliada submetendo as medidas ao coeficiente de correlação de Spearman, que avalia o grau de intensidade existente na relação entre duas variáveis não paramétricas (varia entre -1 e +1, e quanto maior o valor absoluto, mais forte será a relação entre os escores).

Todos os instrumentos analisados apresentaram relação significativa entre si segundo o coeficiente de Spearman ( *p* < 0,05). Entretanto, o nível de correlação foi distinto:

- A cor relação positiva perfeita (com valor de *r* próximo de 1) foi observada entre os instrumentos WOSI x DASH ( *r* = 0,96). Esse resultado indica que as medidas de ambos os indicadores aumentam significativamente na mesma intensidade.
- Além desses, os instrumentos WOSI e UCLA ( *r* = 0,87), DASH e UCLA ( *r* = 0,86), SSV e Rowe ( *r* = 0,80), EVA e DASH ( *r* = 0,75), EVA e UCLA ( *r* = 0,74) e WOSI e EVA ( *r* = 0,72) também apresentaram tendência de linearidade positiva entre as medidas.
- Já os instrumentos WOSI e SSV, WOSI e Rowe, DASH e Rowe, SSV e UCLA ( *r* = -0,83), SSV e DASH ( *r* = -0,79), Rowe e UCLA ( *r* = -0,78), EVA e SSV ( *r* = -0,68) e EVA e Rowe ( *r* = -0,60) apresentaram correlação negativa .

## Discussão

A avaliação e a análise dos resultados de determinados tratamentos na prática médica evoluíram nos últimos tempos. A percepção do paciente sobre seu problema passou a ser mais valorizada no contexto atual. Assim, escores baseados em uma análise mais subjetiva se tornaram os principais métodos de avaliação recomendados.

Existem outros métodos de avaliação para instabilidade do ombro, mais o WOSI foi considerado o melhor entre eles, 7 14 e já foi validado em vários idiomas; 2 4 5 7 15 16 17 18 19 20 21 22 a escolha do método de análise deste trabalho foi baseada nos artigos previamente publicados sobre a validação desse escore.

Os escores foram selecionados devido à possibilidade de comparação com outros estudos de validação do WOSI já realizados, e por englobarem diferentes instrumentos de avaliação, sejam eles genéricos (EVA), específicos do membro (DASH), específicos do ombro (UCLA, SSV) ou específicos para determinada patologia (Rowe). O DASH já foi validado em português brasileiro, 9 e os escores de Rowe e UCLA foram submetidos a tradução e adaptação cultural para português do Brasil. 10 11 A EVA de dor é amplamente utilizada em sua versão em português, a despeito de não haver validação específica publicada. Atribuímos isso ao fato de ela ser extremamente compreensível, por se tratar de escala visual, sem perguntas complexas que dependeriam de adaptação cultural. Da mesma forma, o SSV é uma avaliação subjetiva do paciente expressa em percentuais, e 100% é o valor de referência para o ombro completamente normal.

Todos os pacientes dos grupos pós-operatório e conservador apresentavam instabilidade anterior (para diminuir o viés na interpretação dos resultados). A idade média dos pacientes do estudo (de 27,35 ± 5,43 anos) e o gênero (44 homens [82,36%] e 7 mulheres [17,64%] ) são correspondentes aos de estudos prévios, 4 7 18 e se mostraram homogêneos na comparação entre os grupos ( Tabela 1 ).

A análise da validade do WOSI em português do Brasil, segundo o coeficiente de Spearman, revela correlação significativa boa ou excelente entre todos os escores (apresentam valores próximos a -1 ou 1). Esse coeficiente é interpretado como excelente (> 0,91), bom (0,90–0,71), razoável (0,70–0,51), ruim (0,50–0,31) e nulo (< 0,31). 4

Individualmente, o WOSI apresenta correlação quase perfeita com o DASH (0,96) e alta correlação com o UCLA (0,87) e a EVA (0,72) ( Tabela 3 ). Esses resultados são correspondentes aos encontrados no artigo original (DASH = 0,77; UCLA = 0,65), 2 e na validação para o francês (QuickDASH = 0,78; EVA = 0,83), 4 o turco (DASH = 0,67), 7 o alemão (UCLA = 0,61), 21 o holandês (DASH = 0,81), 20 o sueco (EVA = 0,80), 15 o italiano (DASH = 0,79) 18 e o japonês (QuickDASH = 0,63). 17 Todos esses apresentaram linearidade positiva para tais escores, em especial DASH e UCLA.

**Tabela 3:** Análise de correlação (coeficiente de Spearman) entre as pontuações nos instrumentos utilizados

		WOSI	EVA	SSV	Rowe	DASH	UCLA
**WOSI**	valor de *r*		0,72	-0,83	-0,83	0,96	0,87
	valor de *p*		< 0,0001	< 0,0001	< 0,0001	< 0,0001	< 0,0001
**EVA**	valor de *r*	0,72		-0,68	-0,60	0,75	0,74
	valor de *p*	< 0,0001		< 0,0001	< 0,0001	< 0,0001	< 0,0001
**SSV**	valor de *r*	-0,83	-0,68		0,80	-0,79	-0,83
	valor de *p*	< 0,0001	< 0,0001		< 0,0001	< 0,0001	< 0,0001
**Rowe**	valor de *r*	-0,83	-0,60	0,80		-0,83	-0,78
	valor de *p*	< 0,0001	< 0,0001	< 0,0001		< 0,0001	< 0,0001
**DASH**	valor de *r*	0,96	0,75	-0,79	-0,83		0,86
	valor de *p*	< 0,0001	< 0,0001	< 0,0001	< 0,0001		< 0,0001
**UCLA**	valor de *r*	0,87	0,74	-0,83	-0,78	0,86	
	valor de *p*	< 0,0001	< 0,0001	< 0,0001	< 0,0001	< 0,0001	

**Abreviaturas:** EVA, Escala Visual Analógica; DASH, Disabilities of the Arm, Shoulder and Hand; SSV, Subjective Shoulder Value; UCLA, University of California-Los Angeles; WOSI, Western Ontario Shoulder Instability Index.

Quanto à correlação do escore de Rowe (-0,83), mostrou-se também boa linearidade negativa, semelhante à das análises francesa (-0,54) 4 e turca (-0,57), 7 assim como a do artigos original (0,61), 2 e da validação para o alemão (0,62) 21 e o sueco (0,59), 15 porém com linearidade positiva. Em relação ao SSV (-0,83), foi observada boa linearidade negativa, sem comparação com outros estudos.

Entre os pontos positivos deste trabalho, encontram-se o número de 5 escores analisados (superior ao de outros estudos de validação, que utilizaram 3 ou 4), a homogeneidade dos grupos, a representação da epidemiologia da instabilidade do ombro e a alta correlação significativa entre os escores. Como pontos negativos, o tamanho pequeno, porém significativo, da amostra, e a não realização do teste-reteste e das análises inter e intraobservador.

## Conclusão

Concluímos que o Western Ontario Shoulder Instability Index (WOSI) tem boa validade no português do Brasil.
